# CFMDS: CUDA-based fast multidimensional scaling for genome-scale data

**DOI:** 10.1186/1471-2105-13-S17-S23

**Published:** 2012-12-07

**Authors:** Sungin Park, Soo-Yong Shin, Kyu-Baek Hwang

**Affiliations:** 1 School of Computer Science and Engineering, Soongsil University, Seoul 156-743, Korea; 2 Department of Clinical Epidemiology and Biostatistics, Asan Medical Centre, Korea; 3 University of Ulsan College of Medicine, Seoul 138-736, Korea

## Abstract

**Background:**

Multidimensional scaling (MDS) is a widely used approach to dimensionality reduction. It has been applied to feature selection and visualization in various areas. Among diverse MDS methods, the classical MDS is a simple and theoretically sound solution for projecting data objects onto a low dimensional space while preserving the original distances among them as much as possible. However, it is not trivial to apply it to genome-scale data (e.g., microarray gene expression profiles) on regular desktop computers, because of its high computational complexity.

**Results:**

We implemented a highly-efficient software application, called CFMDS (CUDA-based Fast MultiDimensional Scaling), which produces an approximate solution of the classical MDS based on CUDA (compute unified device architecture) and the divide-and-conquer principle. CUDA is a parallel computing architecture exploiting the power of the GPU (graphics processing unit). The principle of divide-and-conquer was adopted for circumventing the small memory problem of usual graphics cards. Our application software has been tested on various benchmark datasets including microarrays and compared with the classical MDS algorithms implemented using C# and MATLAB. In our experiments, CFMDS was more than a hundred times faster for large data than such general solutions. Regarding the quality of dimensionality reduction, our approximate solutions were as good as those from the general solutions, as the Pearson's correlation coefficients between them were larger than 0.9.

**Conclusions:**

CFMDS is an expeditious solution for the data dimensionality reduction problem. It is especially useful for efficient processing of genome-scale data consisting of several thousands of objects in several minutes.

## Background

Multidimensional scaling (MDS) is a technique for representing objects (or data points) in a low-dimensional space based on their similarity. Main purposes of MDS include exploratory data analysis by visualization and feature selection for subsequent analysis such as classification. In bioinformatics and related areas, MDS has been applied to diverse problems such as gene expression pattern visualization [1,2], drug responses profiling [3], and p53 transactivation prediction [4].

Among various MDS methods, the classical MDS is based on the idea of finding coordinates appropriate for describing dissimilarities as distances [5]. The classical MDS finds coordinates by a set of matrix operations. Roughly speaking, it decomposes the squared distance matrix by solving the eigenpair problem, of which complexity is proportional to the cube of the number of data points [6]. This heavy computational burden is a bottleneck for quick processing of large-scale datasets having thousands of objects. Meanwhile, massive parallel processing based on graphics processing units (GPUs) for general computing applications, a.k.a. GPGPU (general purpose computation on graphics processing units) has risen as a reasonable option for expediting computationally-intensive jobs on normal desktop computers equipped with a graphics card [7]. CUDA (compute unified device architecture) is one of the most pervasively-used frameworks for GPGPU developed by NVIDIA, Inc. [8]. In the CUDA environment, linear algebra packages such as CUBLAS [8] and CULA [9] are provided. In bioinformatics, CUDA has been deployed for diverse applications such as sequence alignment [10-12], protein substructure search [13], RNA microarray analysis [14], and a non-classical MDS [15].

One problem with CUDA is the relatively small memory size of most graphics cards (usually less than 1 gigabyte). General graphics cards do not have sufficient memory for storing and processing large-scale datasets containing tens of thousands data points. For circumventing this problem, we exploit a famous engineering principle, i.e., divide-and-conquer. Divide-and-conquer approach to the classical multidimensional scaling has drawn much attention for reducing its computational complexity and has been applied in serial computing environments [6,16].

## Implementation

We implemented CFDMS by extending our previous work [17]. Our software application has two operating modes. If a graphics card allows sufficient memory for reading and processing all data points, it runs in "one-shot" mode. When available memory is not enough, it operates in "divide-and-conquer" mode and produces an approximate solution. The available memory size is automatically detected and the two operating modes are accordingly toggled on and off.

### One-shot MDS

In the one-shot mode, the classical MDS on a dissimilarity matrix **D**, of which size is *n *× *n*, proceeds as follows.

1. **D**^(2) ^= [*d_ij _*^2^], where *d_ij _*denotes the element of **D **on the *i*th row and the *j*th column, i.e., the dissimilarity between the *i*th and *j*th points.

2. **J **= **I **- *n*^-1^**1**, where **I **is the identity matrix and **1 **denotes the *n *× *n *matrix of which elements are all one.

3.$\text{B}=\;-\;\frac{1}{2}\text{J}{\text{D}}^{\left(2\right)}\text{J}$.

4. Calculate the first *m *eigenvectors **e**_1_, **e**_2_, ..., **e***_m _*and the corresponding eigenvalues λ_1_, λ_2_, ..., λ*_m _*from **B**.

5. Calculate the *m*-dimensional coordinates of the *n *data points by $X^{T}=\left[e_{1},\;e_{2},\;...,\;e_{m}\right]\Lambda_{m}^{\frac{1}{2}},\;\text{where }\Lambda_{m}^{1/2}=\text{diag}\left(\lambda_{1}^{1/2},\;\lambda_{2}^{1/2},\;...,\;\lambda_{m}^{1/2}\right).$

Each column of **X **corresponds to the coordinate of each data point in the reduced (*m*-dimensional) space. The above procedure has been implemented using CUBLAS [8] and CULA [9].

### Divide-and-conquer MDS

**Figure 1:**
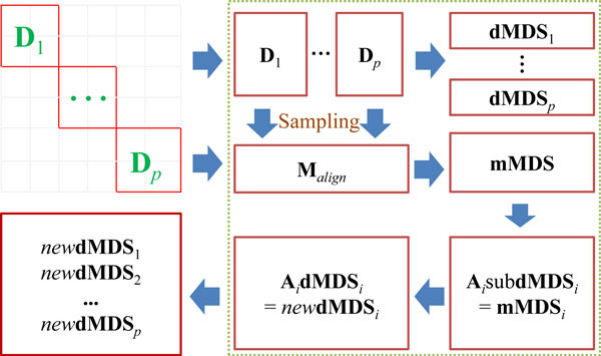
**Process of divide-and-conquer mode**. First, a dissimilarity matrix is randomly decomposed into *p *submatrices along the diagonal, **D**_1_, ..., **D***_p_*. Second, *s *objects are sampled from each of the submatrices. Then, the sampled objects are merged to construct a new dissimilarity submatrix **M***_align_*. The one-shot MDS method is applied to **D**_1_, ..., **D***_p _*as well as **M***_align_*. The resulting coordinates are **dMDS**_1_, ..., **dMDS***_p _*as well as **mMDS**, respectively. After that, the objects sampled from each of **D**_1_, ..., **D***_p _*are extracted from the resulting coordinates matrices, comprising sub**dMDS**_1_, ..., sub**dMDS***_p _*as well as **mMDS**_1_, ..., **mMDS***_p_*. For each pair, sub**dMDS***_i _*and **mMDS***_i _*(*i *= 1, 2, ..., *p*), a linear transformation matrix **A***_i _*is obtained by minimizing ||**A***_i_*sub**dMDS***_i _*- **mMDS***_i_*||, where || · || denotes *L*^2 ^norm. The linearly transformed objects *new***dMDS***_i _*on a reduced dimension are obtained by **A***_i_***dMDS***_i_*. Finally, *new***dMDS**_1_, ..., *new***dMDS***_p _*are combined to produce the MDS result for the entire objects.

The divide-and-conquer MDS based on [6] divides a given set of objects into several subsets of manageable size. Then, another subset of manageable size is made by sampling from each of the previous subsets. The same MDS routine of the one-shot mode is applied to each of the submatrices. Finally, each result is merged into an approximate MDS solution for the entire objects. More precise steps are as follows (see Figure 1).

1. Randomly decompose an *n *× *n *dissimilarity matrix **D***_all _*along the diagonal into *p *submatrices, i.e., **D**_1_, **D**_2_, ..., **D***_p_*.

2. Sample *s *objects from each of the submatrices.

3. Merge the sampled objects and construct a new dissimilarity submatrix **M***_align _*of which size is (*sp*) × (*sp*).

4. Apply the one-shot MDS method to **D**_1_, **D**_2_, ..., **D***_p _*as well as **M***_align_*. Denote the resulting coordinates by **dMDS**_1_, **dMDS**_2_, ..., **dMDS***_p _*as well as **mMDS**, respectively.

5. Extract the objects sampled at step 2 from the above results, obtaining sub**dMDS**_1_, sub**dMDS**_2_, ..., sub**dMDS***_p _*as well as **mMDS**_1_, **mMDS**_2_, ..., **mMDS***_p_*.

6. For each pair sub**dMDS***_i _*and **mMDS***_i _*(*i *= 1, 2, ..., *p*), solve the following linear least squares problem, argmin_**A***i *_||**A***_i_*sub**dMDS***_i _*- **mMDS***_i_*||, where || · || denotes *L*^2 ^norm.

7. Linearly transform the objects of **D***_i _*as follows. **A***_i_***dMDS***_i _*= *new***dMDS***_i_*.

8. Combine *new***dMDS**_1_, *new***dMDS**_2_, ..., *new***dMDS***_p _*into an approximate MDS solution to the entire objects.

Since the size of submatrix is determined by the available memory size of a graphics card, the number of submatrices *p *and the number of sampled objects from each submatrix *s *are determined automatically by our software application. Two ways of sampling from the submatrices (Step 2 of the algorithm above) are "Random" and "MaxMin". *Random *denotes usual random sampling without replacement. In the *MaxMin *approach, data points are chosen one at a time, and each new point maximizes, over all unused data points, the minimum distance to any of the previously-sampled points [18]. As in the one-shot mode, all the matrix operations have been implemented using CUBLAS and CULA.

## Results

CFMDS has been tested using five benchmark datasets. Table 1 describes the data source and simple characteristics of each dataset. As shown in Table 1, diverse datasets, ranging from a simple dataset with four attributes to complicated microarrays and handwritten digits, were used to demonstrate the performance of CFMDS. Experiments were performed using a commodity PC equipped with an Intel Core2 Quad Processor Q6600 (2.4 GHz), 4 GB of RAM, and GeForce 8600 GT (graphics card). The operating system was Windows XP (32-bit version). CFMDS was run on this PC. For comparison, a general solution for the classical MDS was implemented using C# on this computer. However, the C#-based implementation was not able to process *S*. *cerevisiae *Microarray and MNIST datasets due to a memory shortage on the PC (4 GB only). For these large datasets, the classical MDS algorithm was implemented using MATLAB on a 64-bit Linux PC Server equipped with two Intel Xeon Processors E5506 (2.13 GHz) and 32 GB of RAM. It should be noted that the performance of matrix operations in MATLAB are known to be generally better than those implemented by other efficient languages such as C++ [15]. Parameter settings for the experiments are shown in Table 2. The size of dissimilarity matrix is *n *× *n*, where *n *is the number of instances in Table 1. The number of submatrices (*p*) and the number of objects sampled from each submatrix (*s*) were set based on the available memory size of the graphics card for *S. cerevisiae *Microarray and MNIST datasets. For IRIS, Dermatology, and *M*. *musculus *Microarray datasets, these parameters were set arbitrarily because they can be processed by the one-shot mode of CFMDS.

**Table 1:** Benchmark datasets

Dataset	Source	Number of Attributes	Number of Instances	Pearson's Median Skewness Coefficient	Coefficient of Variation
IRIS	UCI ML Repository	4	150	0.34	0.64
Dermatology	UCI ML Repository	33	366	-0.61	0.42
*M. musculus *Microarray	GEO	4,000	2,000	0.94	1.08
*S. cerevisiae *Microarray	GEO	1,000	9,300	0.73	0.56
MNIST	MNIST	784	10,000	-0.13	0.14

UCI ML Repository is UCI Machine Learning Repository http://archive.ics.uci.edu/ml/datasets.html. GEO is Gene Expression Omnibus http://www.ncbi.nlm.nih.gov/geo/. MNIST is the MNIST Database of handwritten digits http://yann.lecun.com/exdb/mnist/. *M*. *musculus *Microarray is a modified dataset from *Mus musculus *microarrays in GEO and *S*. *cerevisiae *Microarray is a modified dataset from *Saccharomyces cerevisiae *microarrays in GEO. MNIST dataset is from scanned handwritten digit images of 28 × 28 pixels.

**Table 2:** Experimental setting

Dataset	Size of Dissimilarity Matrix	No. of Submatrices (*p*)	No. of Samples in Each Submatrix (*s*)
IRIS	150 × 150	3	20
Dermatology	366 × 366	3	60
*M. musculus *Microarray	2,000 × 2,000	10	100
*S. cerevisiae *Microarray	9,300 × 9,300	10	150
MNIST	10,000 × 10,000	10	150

These parameters were set for comparison experiments of the divide-and-conquer mode of CFMDS. In fact, the CFMS application automatically detects the available memory size and these parameters are subsequently determined. For IRIS, Dermatology, and *M. muculus *Microarray datasets, these parameters were set arbitrarily, because they can be processed by the one-shot mode of CFMDS.

### Execution time of CFMDS

**Figure 2:**
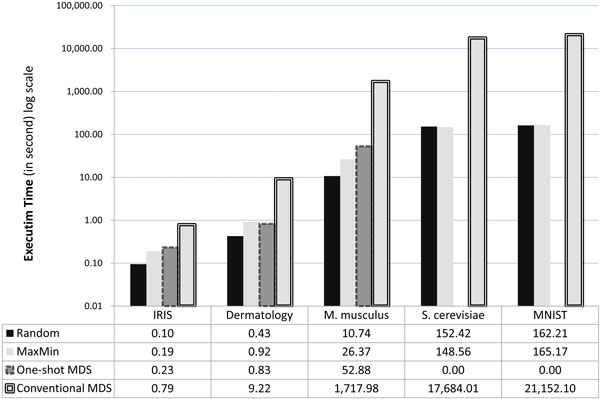
**Comparison results of execution time**. Average running time in seconds is shown. The y-axis is in log scale. Random (MaxMin) means the divide-and-conquer mode of CFMDS with *Random *(*MaxMin*) sampling. One-shot MDS represents CFMDS without divide-and-conquer. Conventional MDS represents the classical MDS implemented using C# or MATLAB in serial computing environments. "0.00" denotes "not applicable." For *S. cerevisiae *and MNIST datasets, we were not able to apply the one-shot mode of CFMDS due to the memory limitation in our graphics card.

The execution time was compared to demonstrate the speed-up of the proposed application. Figure 2 shows the execution time of each method including CFMDS with *Random *sampling, CFMDS with *MaxMin *sampling, one-shot CFMDS, and conventional solutions for the classical MDS in serial computing environments. In the figure, the y-axis is in log scale. As expected, CFMDS showed significant improvement in running time for large datasets such as the two microarray and MNIST datasets. For the most time-consuming dataset, MNIST, the conventional MDS algorithm took almost 6 hours to get the result. However, CFMDS with *Random *or *MaxMin *sampling produced the results from the same dataset within 3 minutes. CFMDS with *Random *sampling was more than 100 times faster than the conventional MDS algorithm for *M. musculus *and *S. cerevisiae *datasets. CFMDS with *MaxMin *sampling was more than 66 times faster than the conventional MDS algorithm for these microarray datasets. CFMDS also achieved significant speed-up for even small datasets such as IRIS and Dermatology, ranging from 3 to 22 times faster. These results confirm the fact that the proposed application is very useful for fast multidimensional scaling of diverse datasets, not only of genome-scale data. We also verified the necessity of our divide-and-conquer strategy for large data. Both the one-shot and divide-and-conquer modes of CFMDS required similar computational time for small datasets such as IRIS and Dermatology. However, the one-shot mode needed much more computational time than the divide-and-conquer mode for *M*. *musculus *Microarray dataset. Further, the one-shot mode was not able to process *S*. *cerevisiae *Microarray and MNIST datasets due to the limitation of memory in the graphics card. "0.00" in Figure 2 means "not applicable."

### Accuracy of CFMDS

**Figure 3:**
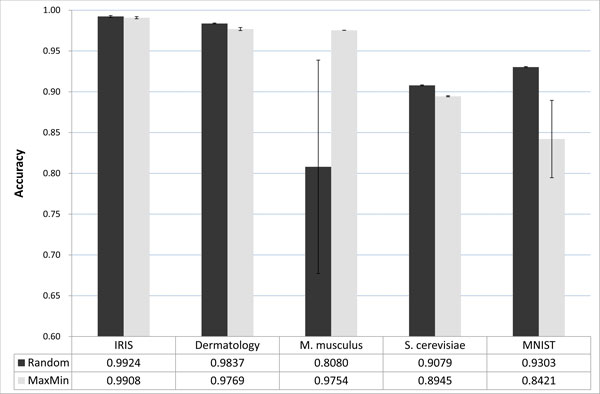
**Comparison results of accuracy**. Pearson's correlation coefficient was used as accuracy. The mean value and standard deviation from 100 independent simulation results are shown. Random (MaxMin) means the divide-and-conquer mode of CFMDS with *Random *(*MaxMin*) sampling.

To examine the accuracy of the divide-and conquer mode of CFMDS, Pearson's correlation coefficient between the results from the classical MDS and CFMDS was used. More precisely, vectors, consisting of the Euclidean distance between each object pair on a reduced dimension, were generated from the results of the classical MDS and CFMDS, respectively. Then, Pearson's correlation coefficient between these vectors was calculated. As the correlation coefficient is close to 1, the result from the divide-and-conquer mode of CFMDS is similar to the result from the classical MDS. The accuracy comparison results are shown in Figure 3. The figure depicts average values of 100 independent runs with error bars representing standard deviation. As shown in Figure 3, the divide-and-conquer mode of CFMDS produced highly accurate results from all datasets. Pearson's correlation coefficients were larger than 0.9 in *Random *or *MaxMin *samplings. For the simplest IRIS dataset, which has 4 attributes and 150 instances, CFMDS achieved almost identical results compared to the classical MDS (Pearson's correlation coefficient: about 0.99) both in *Random *and *MaxMin *sampling modes. Dermatology and *S. cerevisiae *Microarray datasets showed similar trends with decrease in accuracy compared to the IRIS dataset.

However, CFMDS with *Random *and *MaxMin *sampling modes showed different results for *M. musculus *Microarray and MNIST datasets. For *M. musculus *Microarray dataset, *Random *sampling mode showed the worst result among all benchmark datasets with the largest standard deviation, although *MaxMin *sampling method produced almost identical results compared to the result from the classical MDS (Pearson's correlation coefficient: about 0.97). On the contrary, *MaxMin *mode showed a relatively low performance with high variance for MNIST dataset. For the same dataset, *Random *sampling mode achieved relatively accurate results (Pearson's correlation coefficient: about 0.93). The difference in performance of *Random *and *MaxMin *sampling methods of CFMDS could be due to the skewness or dispersion of data. The *MaxMin *sampling mode is suitable for datasets with high skewness or dispersion, because it could sample data points which are far apart from each other [18]. We checked the skewness and dispersion of our experimental datasets using Pearson's median skewness coefficient and coefficient of variation of distances between data points. The Pearson's median skewness coefficient (PMSC) is defined as 3(*mean *- *median*)/*standard deviation *and measures asymmetry of a distribution. Coefficient of variation (CV) is defined as *standard deviation */*mean *and is a normalized measure of dispersion. Among the five datasets, *M. musculus *Microarray showed the highest skewness and dispersion (PMSC = 0.94, CV = 1.08). For this dataset, *MaxMin *sampling mode of CFMDS generated relatively accurate results. MNIST dataset showed the lowest skewness and dispersion (PMSC = -0.13, CV = 0.14). For this dataset, *Random *sampling mode showed relatively accurate results. As a conclusion, we suggest the use of *MaxMin *sampling for highly skewed or dispersed data and *Random *sampling for symmetric and lowly dispersed data.

## Discussion

We implemented a software application, CFMDS (CUDA-based Fast MultiDimensional Scaling) for efficient dimensionality reduction of large-scale genomic data. CFMDS adopted CUDA programming library and divide-and-conquer strategy to handle several thousands of features in less than several minutes on a commodity PC equipped with a graphics card. CUDA was applied as a parallel computing method and divide-and-conquer principle was used to circumvent the small memory size problem of usual graphics cards. By combining these two techniques, CFMDS enables that a regular PC with a CUDA-support graphics card handles the large-scale genomic data dimensionality reduction problem which can be efficiently executed only on high performance computers. The simulation results confirmed that our approach can perform MDS more than a hundred times faster with a comparable accuracy for genome-scale data. Therefore, CFMDS is especially useful to visualize and analyze data consisting of several thousands of objects in less than several minutes. We implemented two sampling options for the divide-and-conquer mode of CFMDS such as *Random *and *MaxMin *samplings. As shown in Results section, CFMDS with *Random *sampling approach usually works quite well in practice. *MaxMin *sampling method is especially useful in some contexts where data distribution is highly skewed or dispersed. Further work includes optimizing our application with respect to data transfer between graphics cards and host computers.

## Availability and requirements

Project name: CFMDS

Project home page: http://ml.ssu.ac.kr/CFMDS/CFMDS.html

Operating system(s): Windows XP or higher (32-bit and 64-bit), Linux (tested on Ubuntu Linux 9.04, Red Hat Enterprise Linux 5.3/4.7, Fedora 11)

Programming language: CUDA

Other requirements: NVIDIA's GPU with CUDA, CUDA toolkit 2.3 (not support CUDA 3.0 toolkit yet), The latest version of CULA basic libraries

License: GNU GPL v2

Any restrictions to use by non-academics: none

## Competing interests

The authors declare that they have no competing interests.

## Authors' contributions

S.P. developed the software application and performed the experiments. S.-Y.S. wrote the manuscript and discussed the results. K.-B.H. led the project and wrote the article. All of the authors have read and approved the final manuscript.
